# Pesticides pollution and risk assessment of river Ganga: A review

**DOI:** 10.1016/j.heliyon.2021.e07726

**Published:** 2021-08-08

**Authors:** Zeshan Umar Shah, Saltant Parveen

**Affiliations:** Limnology Research Laboratory, Department of Zoology, Aligarh Muslim University, Aligarh, 202002, India

**Keywords:** Pesticides, River Ganga, Risk assessment, Future prospect

## Abstract

Pesticides consumption along with its impact on different matrices of the environment has increased over past. Monitoring and risk assessment is important to know the exact scenario of pesticide toxicity of aquatic environment. The article compiles the number of studies on different stretches of river Ganga over past years. Risk quotient (RQ) method was used for the determination of potential risk of reported pesticides. Based on general (RQm) and worst-case (RQex) DDT and aldrin in the middle stretch of river Ganga show high risk. Regular monitoring along with compartmental studies is important to assess the pesticide pollution load and persistence in the river. Because hundreds of formulations are being used in the basin for agricultural purposes, detailed analysis and bio-magnification of all the pesticides should be appreciated.

## 1 Introduction

The Ganga river basin spreads over 860,000 km^3^ i.e., actually 26.3 % of the total geographical area of the country and accommodates over 43% of the country population. The basin stretches over eleven states from Uttrakhand, Himachal Pradesh, Haryana, Delhi, Uttar Pradesh, Bihar, Jharkhand, Rajasthan, Madhya Pradesh, Chhattisgarh and West Bengal. River Ganga the second largest river supports 36.1% of the total population of the country by agriculture and other means (Census, 2011). From the origin of the river to the outfall, it is supported by number of tributaries to form very huge and highly productive indo-Gangetic, which gives rise to exhaustive agricultural activities in the basin.

The bio-ecological functions of the river Ganga have been highly changed over past by large scale anthropogenic activities such as industrial discharge and domestic wastes, dams and barrages, agro-fertilizers used in agricultural areas, pesticides and insecticides. High pesticide application along the river basin agricultural fields let to the bioaccumulation of these residues in the ecosystem through several means (Schulz and Peall, 2001; Gavrilescu, 2005; Holvoet et al., 2007; Takatori et al., 2008), accumulation of these residues has the potential of impact on non-target organisms, and to the natural environmental conditions (Takatori et al., 2008; Carriquiriborde et al., 2014). The total usage of pesticides in Ganga basin between year 2012–2017 was 72,741 MT, which is 27% of countries total consumption (PQRS, 2017). To the best of our knowledge this is the first attempt to sum up all the studies done on the river to give comprehensive status of pesticide pollution in the river. Previous studies carried out have shown organochlorine pesticides being abundantly used, as detected in number of studies carried out (Rehana et al., 1995; Sankararamakrishnan et al., 2005; Ghosh et al., 2009; Singh et al., 2012; Mutiyar and Mittal 2013; Chakraborty et al., 2016; and Mondal et al. 2018).

These pesticides, organochlorines, organophosphates, pyrethroids and carbamates upon application cause serious impact by their persistence in the environment, toxicity to non-target organisms, long-range transmission, and bio-accumulation under favorable environmental conditions (Naqvi and Vaishnavi, 1993; Contreras López, 2003; Briz et al., 2011; Gao et al., 2013). Various studies have shown incidence of various cancers in human tissues (Mathur et al., 2002; Rathore et al., 2002; Abdo et al. 2013; Ennour-Idrissi et al., 2019),teratogenicity (Kalra et al., 2016; Kim et al., 2017; Ramakrishnan et al., 2019), endocrine dysfunctioning (Fowler et al., 2007; Frye et al., 2012), nerve dysfunctioning (Shinomiya and Shinomiya, 2003; Sharma et al., 2010; Heusinkveld and Westerink, 2012), and genotoxicity (Ramirez and Cuenca, 2002; Ennaceur et al., 2008). Similarly, wildlife organisms on exposure to pesticides have shown developmental deformities in genitalia (Sonne et al., 2006), abnormal reproductive behavior (Fry, 1995), sterility, cancers, egg-shell thinning, and immune dysfunctioning (Colborn and Smolen, 1996; Briz et al., 2011; Newton, 2013). In year 2006 Stockholm Convention, classes of organochlorine pesticides banned by several countries were still manufactured and used in the country. India the only major country of world has allowed manufacture and use of DDT for vector killing until 2022. The country also supplies DDT for use to several countries for agricultural purposes or as intermediate chemical (Toxics Link, 2018). Manufacture, import, and use of hexachlorocyclohexane and lindane were banned in year 2011. However, there is no restrictions on the use of α-, β-, and δ-HCHs (PPQS, 2019). Production, use and export of endosulfan was allowed till 2011, however recent data have shown exportation of endosulfan still occurs on large scale.

Despite the major plans to control the pesticide pollution, occurrence and high level of pesticides have been continuously detected across many rivers of the country. Hence the regular investigation of the pesticide status in aquatic ecosystem is primary source of information to reframe the policy of manufacture and use. The detected concentration of the pesticides act as indicator of load and frequency of anthropogenic activities along the basin. Besides regular monitoring, ecological risk assessment, spatial and temporal, hotspot of usage is lacking. The lack of intensive studies has already affected eco-biological conservation and management efforts of the Ganga river. Simultaneously research should focus on information collection from all sources to detect pesticide concentration and status of pesticides usage along all five river Ganga states. In the article we aim to sum up the pesticides investigations done along all stretches along with ecological risk assessment. The results will be helpful in knowing the status of pesticide pollution, ecological risk and thus in policymaking for pollution control. This in turn will be helpful in designing the effective monitoring strategies that would result in effective pesticide mitigation efforts for healthy eco-biology of the Ganga river.

## 2 Source of pesticide pollution

**Figure 1:**
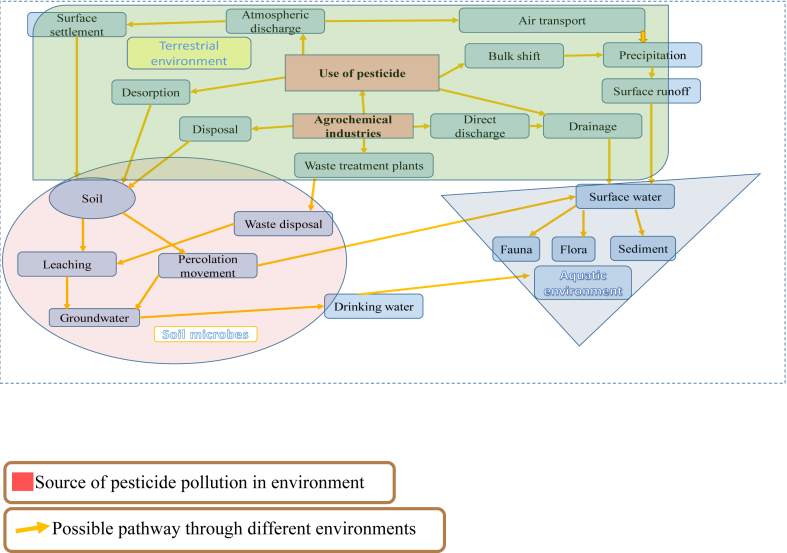
Possible pathway of pesticide pollution in water through different compartments.

Several hundred formulations of pesticides are currently used for agricultural purposes along the river Ganga basin. Estimated from agricultural and other sources, above 9000 MT of pesticides were annually used for agricultural practices in the Ganga river basin (Ghosh et al., 2009). In the recent studies of (NGRBA, 2011) it is estimated 21000 MT of pesticides are applied in the basin annually. The pattern of use shows insecticides always dominate the formulations followed by herbicides and at last fungicides. In the insecticide category, organochlorines were predominantly used till 1990, after which the use of organophosphate formulations increased. The consumption pattern of pesticide uses in year 2014 was, insecticide 80%, followed by herbicide 15% and fungicide 2%. Phosphamidon, butachlor, mancozeb, quinalphos, monocrotophos, paraquat, endosulfan, isoproturon are commonly consumed pesticides (Bhushan et al., 2003). Due this enormous quantity of use possibilities of pesticide transport to the aquatic ecosystem increases by surface runoff, leaching and flash floods. Applied pesticides in the agricultural fields may be present in the rain water because of their volatilization from soil and crops. Although rainwater is considered as safe and fit for use, the source is also polluted by pesticides as reported by (Sakai 2002; Kumari et al., 2007). Figure 1 depicts the source and path of pesticide occurrence in water.

## 3 Literature survey

The present review article amid to provide the status of pesticide pollution in the river Ganga based on the total number of studies done along the different stretches. The literature cited in the present study was extracted using following keywords river Ganga, pesticides, agriculture and water pollution. More than six publications were reviewed compiling thirteen stations along the whole length of river Ganga. The data was uniformly converted in the same units of (ng/l) for ease of reading and understanding. Organochlorines are the compounds that persist for longer duration than other formulations and have severe impact on the environment. The total number of studies along the length of river Ganga has mainly focused on the organochlorines. Based on survey of literature as no study has been carried out on the other class of pesticides therefore in the article our main focus will be on organochlorines pesticides.

## 4 Residues in river Ganga water

Liquid-liquid extraction followed by gas chromatography mass spectrometry method was used for the analysis of pesticides in water samples collected from each station. At Champanala, Mond ghat and Burning ghat stations (Singh et al., 2012), among the detected compounds o, p-DDT (489.0 ng/l) and α-endosulfan (168.09 ng/l) were detected in highest concentration at champanala. At Mond ghat α-endosulfan and β-endosulfan (739 ng/l, 157.30 ng/l) and lindane (74.04 ng/l) were found in the highest concentration and at Burning ghat α-endosulfan, o, p-DDT, and p, p′-DDT were reported in highest concentration of (145 ng/l, 125.0 ng/l and 112.0 ng/l) similarly. Lindane was reported in lower concentration as compared to other detected pesticides. The reported level of DDT was quite high; reason could be continuous use or probable slow degradation due to favorable conditions. Endosulfan was also found high in all the collected samples along the stretches.

Water samples were collected from three stations Uttrakhand, Uttar Pradesh and Bihar of river Ganga and analyzed for pesticide residues (Mutiyar and Mittal, 2013). In the total of 82 analyzed samples 16 major pesticides were detected covering endosulfan, HCH, aldrin, heptachlor and DDT. In the upper stretch HCHs were the dominant detected pesticide; endosulphan's were mostly detected in samples collected from middle stretch where as aldrin dominated the list in the lower stretch. Pesticide concentrations reported at Devprayag station were 7.24 ng/l of Σ HCH, 2.3 ng/l Σ aldrin, and 0.07 ng/l Σ heptachlor; at Rishikesh station, 5.54 ng/l ΣHCH, 1.01 ng/l ΣDDT, 0.92 ng/l Σ endosulphan, 1.89 ng/l Σ aldrin and 0.32 ng/l Σ heptachlor, at Haridwar station, 5.2 ng/l ΣHCH, 0.19 ng/l ΣDDT, 0.16 ng/l Σ endosulphan, 0.12 ng/l Σ aldrin, and 0.06 ng/l Σ heptachlor; at Kannauj station, 1.0 ng/l ΣHCH, 0.12 ng/l ΣDDT, 31.66 ng/l Σ endosulphan, 1.3 ng/l Σ aldrin, and 0.2 ng/l Σ heptachlor; at Kanpur station, 0.36 ng/l ΣHCH, 0.2 ng/l ΣDDT, 11.6 ng/l Σ endosulphan, 1.1 ng/l Σ aldrin, and 0.08 ng/l Σ heptachlor; at Allahabad station, 3.5 ng/l ΣHCH, 2.21 ng/l ΣDDT, 0.15 ng/l Σ endosulphan, and 0.4 ng/l Σ aldrin; at Varanasi station, 0.7 ng/l ΣHCH, 1.9 ng/l ΣDDT, 85.4 ng/l Σ endosulphan, 2.2 ng/l Σ aldrin, and 0.1 ng/l Σ heptachlor; at Patna station, 5.0 ng/l ΣHCH, 5.03 ng/l Σ endosulphan, and 1.17 ng/l Σ aldrin; and at Bhagalpur station, 17.6 ng/l ΣHCH, 12.3 ng/l ΣDDT, 17.9 ng/l Σ endosulphan, 16.4 ng/l Σ aldrin, and 11.8 ng/l Σ heptachlor were found in the samples.

Spatial and temporal analysis of (Sah et al., 2020) along the five zones of river Ganga for the determination of pesticide residues. Forty-three sampling stations were used along the length of river Ganga for the detailed analyses of banned pesticides pollution dynamics. The selection was done on the bases of agricultural and industrial hotspots. In the post monsoon studies γ-HCH 1722 ng/l was detected in the highest concentration. The other pesticides reported were α-HCH 432 ng/l, β-HCH 24 ng/l, δ-HCH 88 ng/l, p,p’-DDT 14 ng/l, p,p’-DDD 9 ng/l, p,p’-DDE 70 ng/l, t-chlordane 86 ng/l, c-chlordane 18 ng/l, methoxychlor 7 ng/l, α-endosulfan 5 ng/l, β-endosulfan 8 ng/l and endosulfan sulfate 7 ng/l. In the post winter season analysis, 233 ng/l of γ-HCH was reported and was quite high, the other pesticides were α-HCH 108 ng/l, β-HCH 98 ng/l, δ-HCH 58 ng/l, p, p’-DDT 42 ng/l, p, p’-DDD 34 ng/l, p, p’-DDE 62 ng/l, t-chlordane 36 ng/l, c-chlordane 36 ng/l, methoxychlor 15 ng/l, α-endosulfan 4 ng/l, β-endosulfan 25 ng/l and endosulfan sulfate 13 ng/l. Distribution of pesticide residues was non-normal among the different stations therefore, Kruskal-Wallis (KW) with Dunn's post-hoc was applied to test the significant difference. The difference was considered significant at p < 0.05.

In the middle stretch at Varanasi area water samples were collected between Ramnagar and Rajghat for the analysis of pesticide residues (Nayak et al., 1995). At Kedar ghat the concentration of pesticides was o, p'-DDT 44908 ng/l, p, p'-DDT 79818 ng/l, p, p'-DDE 18500 ng/l, α-endosulfan 48828 ng/l, β-endosulfan 17688 ng/l and γ-HCH 15806 ng/l. At Chousathi ghat the concentration of pesticides was o, p'-DDT 30013 ng/l, p, p'-DDT 5085 ng/l, p, p'-DDE 14315 ng/l, α-endosulfan 3928 ng/l, β-endosulfan 15348 ng/l, α-HCH 1783 ng/l, β-HCH 1186 ng/l, γ-HCH 856 ng/l and δ-HCH 583 ng/l. At Mir ghat the concentration of pesticides was o, p'-DDT 13231 ng/l, p, p'-DDT 60000 ng/l, p, p'-DDE 7024 ng/l, α-endosulfan 20803 ng/l and β-endosulfan 8175 ng/l. At Ramnagar (Sepal ghat) the concentration of pesticides were o, p'-DDT 22768 ng/l, p, p'-DDT 3684 ng/l, p, p'-DDE 11092 ng/l, α-endosulfan 25768 ng/l, β-endosulfan 11258 ng/l α-HCH 120 ng/l, γ-HCH 196 ng/l and δ-HCH 526 ng/l. At Rajendra Prasad ghat concentration of pesticides was o, p'-DDT 14944 ng/l, p, p'-DDT 1842 ng/l, p, p'-DDE 4842 ng/l, α-endosulfan 1398 ng/l, β-endosulfan 4886 ng/l, α-HCH 12693 ng/l, β-HCH 36354 ng/l, γ-HCH 14415 ng/l and δ-HCH 36354 ng/l. At Panch Ganga ghat concentration of pesticides was o, p'-DDT 15507 ng/l, p, p'-DDT 7126 ng/l, p, p'-DDE 1866 ng/l, α-endosulfan 38957 ng/l, β-endosulfan 10723 ng/l and α-HCH 1428 ng/l.

Surface water samples were collected from the urban-suburban transects and discharge points of lower stretch of river Ganga were analyzed for pesticide residues (Khuman and Chakraborty 2019). At discharge point concentration of detected pesticides were p, p’ DDE 13 ng/l, p, p’ DDT 41 ng/l, α- HCH 46 ng/l, β- HCH 35 ng/l, γ-HCH 123 ng/l, δ- HCH 65 ng/l, α-Endosulfan 24 ng/l, β- Endosulfan 17 ng/l, Endosulfan Sulfate 13 ng/l, Aldrin 90 ng/l, Heptachlor 519 ng/l and Heptachlor Epoxide 398 ng/l. At urban point pesticides were detected in the concentration of p, p’ DDE 2 ng/l, p, p’ DDT 1 ng/l, α- HCH 1 ng/l, β- HCH 3 ng/l, γ-HCH 1 ng/l, δ- HCH 1 ng/l, α-Endosulfan 20 ng/l, β- Endosulfan 13 ng/l, Endosulfan Sulfate 2 ng/l, Aldrin 11 ng/l, Heptachlor 7 ng/l and Heptachlor Epoxide 12 ng/l. At sub-urban point pesticides were detected in the concentration of p, p’ DDE 1 ng/l, p, p’ DDT 3 ng/l, α- HCH 1 ng/l, β- HCH 2 ng/l, γ-HCH 1 ng/l, δ- HCH 1 ng/l, α-Endosulfan 21 ng/l, β- Endosulfan 1 ng/l, Endosulfan Sulfate 2 ng/l, Aldrin 7, Heptachlor 8 ng/l and Heptachlor Epoxide 14 ng/l. The generated data was subjected to statistical analysis using t-test and ANOVA and was found significant at p < 0.05.

Surface water samples were collected from different stretches of lower Ganga for the determination of organochlorine pesticides in post-monsoon, winter, and summer seasons (Kumari et al., 2001). At Patna upper stretch in the monsoon season HCH 1160 ng/l, DDT 773 ng/l, aldrin 286 ng/l, and endosulfan 14 ng/l. In the summer season HCH 2200 ng/l, DDT 1080 ng/l, aldrin 79 ng/l and endosulfan 69 ng/l. In the winter season HCH 2590 ng/l, DDT 1336 ng/l, aldrin 89 ng/l and endosulfan 0.068 μg/l. At Mokama station in the monsoon season HCH 916 ng/l, DDT 376 ng/l, aldrin 108.9 ng/l, and endosulfan 30 ng/l. In the summer season HCH 2200 ng/l, DDT 1080 ng/l, aldrin 79 ng/l and endosulfan 69 ng/l. In the winter season HCH 1270 ng/l, DDT 837 ng/l, aldrin 369 ng/l and endosulfan 92 ng/l. At Rajmahal station in the monsoon season HCH 661 ng/l, DDT 337 ng/l, aldrin 128 ng/l, and endosulfan 66 ng/l. In the summer season HCH 1200 ng/l, DDT 799 ng/l, aldrin 350 ng/l and endosulfan 80 ng/l. In the winter season HCH 977 ng/l, DDT 374 ng/l, aldrin 182 ng/l and endosulfan 34 ng/l. At Buxar station in the monsoon season HCH 663 ng/l, DDT 291 ng/l, aldrin 167 ng/l, and endosulfan 430 ng/l. In the summer season HCH 1240 ng/l, DDT 285 ng/l, aldrin 117 ng/l and endosulfan 29.8 ng/l. In the winter season HCH 1240 ng/l, DDT 240 ng/l, aldrin 119 ng/l and endosulfan 32 ng/l. The mean concentration of the pesticide residues in different seasons does not vary. Therefore ANOVA was used to test the hypothesis resulted its acceptance at 5% level of significance. The details of the pesticides studies are compiled in Table S1–S6 (supplementary).

**Figure 2:**
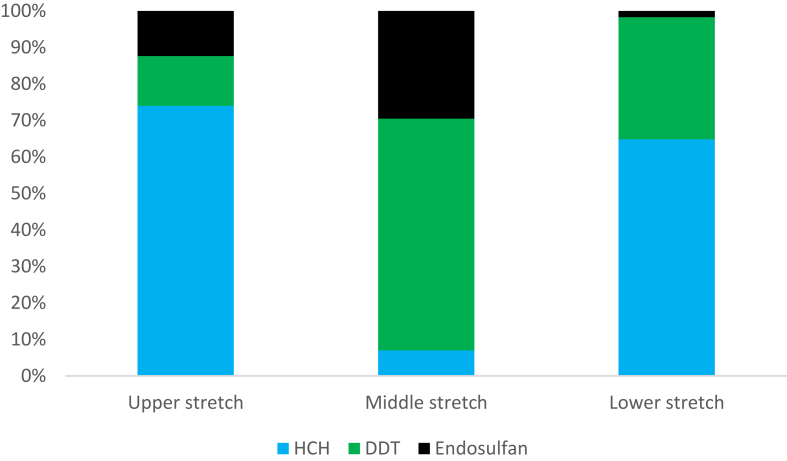
Change in pesticide trend along three stretches of river Ganga.

Figure 2 shows the change in trend of pesticides along the three stretches of river Ganga. In the upper stretch the concentration of HCH is found highest among all the pesticides detected. In the middle stretch i.e., in the Utter Pradesh state DDT is found highest among all the detected pesticides and the concentration of all the pesticides were highest among all the stretches. In the lower stretch of river Ganga HCH was found in highest concentration followed by DDT and endosulfan. Middle stretch receives enormous quantities of pesticides annually for agricultural purposes as the basin is highly fertile and productive.

## 5 Ecological risk assessment

The ecological risk assessment of detected pesticides is calculated by risk quotient (RQ) method (Palma et al., 2014) using the above given data by the equation.

$$\;\text{RQ }=\;\frac{MEC}{PNEC}$$

**Figure 3:**
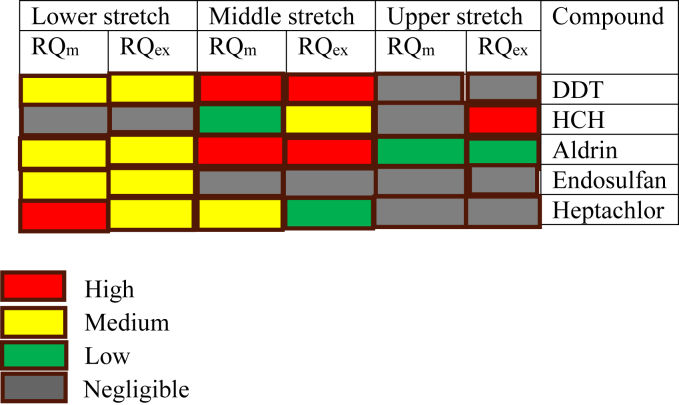
The risk to aquatic species, based on risk ratios, was subsequently classified into four risklevels comprising high, medium, low, and negligible ecological risks, corresponding to RQ values ≥1, 0.1–1, 0.01–0.1, and <0.01, respectively.

MEC is maximum or mean detected concentration of pesticides and PNEC is the predicted no-effect concentration. PNEC is calculated from the toxicity value (i.e., no-observed effect concentration (NOEC) or LC 50 value) of sensitive species of the compartment. In the risk assessment study the respective NOEC value of the pesticide for three trophic levels i.e., primary producer (algae), primary consumer (aquatic invertebrates), secondary consumers (fish) were calculated to determine the PNEC values. The data for Eco-toxicology were obtained from Pesticides Properties Data Base (PPDB) with assessment factor 10 used based on inter and intra-species sensitivity variation. The maximum and mean detected concentration of pesticides were used for calculation of general (RQm) and the worst-case (RQex) scenario. The risk ratio results were classified into four risk levels based on (Palma et al., 2014; Zheng et al., 2016) negligible risk (RQ < 0.01), low risk (0.01 – RQ < 0.1), medium risk (0.1 – RQ < 1) and high risk (RQ ≥ 1). Ecological risk assessment of the studies pesticides is depicted in Table 1 and Figure 3. In the middle stretches of river Ganga DDT and aldrin shows high risk i.e., RQs >1 for both general (RQm using mean MEC) and worst cases (RQex using maximum MEC). Meanwhile, high risk in the worst-case scenario was observed for HCH in upper stretch and from heptachlor in lower stretch. Although the use of persistent pesticides has been banned in the country years ago, still by different trade name the use continues along different agricultural areas. Aldrin and heptachlor are registered for agricultural use and continues for use in abundant quantities. Toxicity assessment by source exposure (pesticide) are important for risk mitigation, legislation and policy making. In this context the concern is inadequate data and knowledge gap of used chemical compositions along the river basin. The selection of risk assessment calculations depends upon the data availability, assessment approach and the type of assessment test (Geissen et al., 2015). Toxicological and Eco-physiological effects (combined or synergistic) of pesticides can be a matter of concern (Johns et al., 2012). Furthermore, the research on the potential combinatorial effects of pesticides is required. The lack of risk assessment has already affected environment monitoring and management efforts. Thus having impact on water quality, aquatic organisms and public health in general. Besides environmental quality risk assessment is vital in designing of standards and guidelines for effective decision making for different compartments.

**Table 1:** Eco-toxicity endpoints for fish, aquatic invertebrates, and algae and RQ evaluation for pesticides in surface water along three stretches of the river Ganga.

Compound	Stretch	NOEC μg/l				Assessment factor	PNEC μg/l	Concentration μg/l		RQ_m_	RQ_ex_
		Fish	Aquatic invertebrates	Algae	Critical concentration			Mean	Maximum		
DDT	Lower	2500	5		5	10	0.5	0.054	0.192	0.108	0.384
	Upper							0.00019	0.00101	0.00038	0.00202
	Middle							9.9	20.6	19.8	41.2
HCH	Lower	2900	54000	1900	1900	10	190	0.269	1.157	0.0014	0.0061
	Upper							0.0055	0.00724	0.0003	3.84
	Middle							5.2	24.5	0.027	0.129
Aldrin	Lower	4.6	28		4.6	10	0.46	0.09	0.291	0.196	0.63
	Upper							0.00189	0.0023	0.0042	0.005
	Middle							10.4	13.4	22.61	29.130
Heptachlor	Lower	7	42	27	7	10	0.7	0.519	2.138	0.742	3.05
	Upper							0.00007	0.00032	0.000001	0.00046
	Middle							0.06	0.2	0.086	0.286
Endosulfan	Lower	2	440	2150	2	10	0.2	0.054	0.106	0.27	0.53
	Upper							0.00016	0.00092	0.0008	0.0046
	Middle							0	0	0	0

## 6 Incompetency of management

Government of India (GOI) has passed several management and action project over past four decades to protect the Ganga river. The first action plan i.e., Ganga action plan (GAP) was developed by government of India in year 1985, which objective was to reduce the pollution level to an acceptable level thus to improve the water quality of this mighty river. However, with all modification along the years in GAP, water quality of the river continued to degrade. The audit report of GAP combined by the performance of the participating states reveals, the project has primarily met 39% of its target. In year 2009 National Ganga River Basin Authority (NGRBA) by the failure of GAP took more firm and compendious basin wise and compartmental steps to ensure effective pollution desisting and conservation of the river by developing a well-planned approach of the management. In year 2014 GOI announced most ambitious action plan Namami Ganga 2014 with the budget of $3 billion USD or more. The objective of the project is to improve water quality with continuous management of the flowing wastes thus to maintain the ecological and biological integrity of the river Ganga. Large quantities of pesticides are used annually along the river basin agricultural areas. By the surface runoff, floods, monsoon rains the left out residues are entering the river in large amount annually. Further by favorable conditions like low temperature these residues persist and tend to bio-concentrate in the food web. Studies have shown residues sometimes are present above the acceptable limit. Thus contributing to the degradation of the water quality of the river. For the achievement of clean Ganga, project should focus on the implementation of its all clauses. For example, lowering the use of pesticides consumption along the basin and effective treatment of the surface runoff waters by use of effective tools like Common Effluent Treatment Plant's (CETPs), enforcement of limit of agrochemical use, regulation for dose recommendation and even long-term shift for organic farming help in reducing water quality deterioration. Areas with high priority and pollution hotspots should be identified by ecological and human health risk assessment. As recontamination of the river is expected and pose potential risks, then long-term change in human behavior and interaction with the mighty river needs to change. Respect for the Ganga river is needed by everyone.

## 7 Future prospect

Although several studies are being carried out to assess the pesticide pollution level along different stretches of river Ganga still the well documented and organized monitoring plan based on knowledge gap is required that includes the following points.

1. Hundreds of different chemical classes are being used along the river Ganga basin, only persistent pesticide chemical class studies are being done by priority. This makes the knowledge gap of other class of pesticides levels and impacts on the aquatic environment.
2. Combined studies should be carried on the different compartments including sediment, water and aquatic biota so that more detailed information of pesticide levels among the environmental compartments are documented.
3. Focus should be on the spatial and temporal analysis of pesticide as during the monsoon more pesticides are added to the water due to flashfloods and surface running.
4. Food web studies are important to get the accurate information of pesticide bio-magnification in the trophic level.
5. Importantly the monitoring and documentation of different class of pesticide used along the basin should be done periodically.

## 8 Conclusion

Based on the number of pesticide studies done along different stretches of river Ganga ecological risk assessment was calculated to monitor the level of pollution toxicity. The risk assessment was assessed based on the bases of deterministic approach, RQ method. The results indicate aldrin and DDT in the middle stations of river Ganga shows high level of risk. While RQex shows high level of risk of HCH in the upper and of heptachlor in the lower stretch. Risk assessment is important for management, policy projects, decision making and their implementations. For the future more detailed and comprehensive studies of total pesticide use along the agricultural areas of river Ganga basin is required. Impact and accumulation of pesticide level in biota along with matrix studies will be highly appreciated and useful.

## Declarations

### Author contribution statement

Zeeshan Umar Shah: Conceived and designed the experiments; Performed the experiments; Analyzed and interpreted the data; Contributed reagents, materials, analysis tools or data; Wrote the paper

Saltant Parveen: Conceived and designed the experiments; Analyzed and interpreted the data; Contributed reagents, materials, analysis tools or data

### Funding statement

This research did not receive any specific grant from funding agencies in the public, commercial, or not-for-profit sectors.

### Data availability statement

Data included in article/supplementary material/referenced in article.

### Declaration of interests statement

The authors declare no conflict of interest.

### Additional information

No additional information is available for this paper.

## Appendix A Supplementary data

supplimentery.docx supplimentery.docx
