# Supplementary material for: Pesticides pollution and risk assessment of river Ganga: A review
Source: Heliyon. 2021 Aug 8;7(8):e07726. doi: 10.1016/j.heliyon.2021.e07726 (PMC8367800; doi:10.1016/j.heliyon.2021.e07726)
Supplement: supplimentery.docx [file mmc1.docx]

| Compound  Site | o, p’-DDT | p,p’-DDT | α-endosulfan | β- endosulfan | Methyl parathion | Lindane |
| --- | --- | --- | --- | --- | --- | --- |
| Champanala | 489.0 | ND | 168.09 | ND | ND | ND |
| Mond ghat | ND | ND | 739.0 | 157.30 | ND | 74.04 |
| Burning ghat | 125.0 | 112.0 | 145.0 | ND | ND | ND |

Table S1. Pesticides reported from Champanala, Mond ghat and Burning ghat of river Ganga.

| Compound  Site | Ʃ HCH | Ʃ DDT | Ʃ endosulfan | Ʃ aldrin | Ʃ heptachlor |
| --- | --- | --- | --- | --- | --- |
| Devprayag | 7.24 | ND | ND | 2.3 | 0.07 |
| Rishikesh | 5.5 | 1.01 | 0.92 | 1.89 | 0.06 |
| Haridwar | 5.2 | 0.19 | 0.16 | 0.12 | 0.06 |
| Kannauj | 1.0 | 0.12 | 31.6 | 1.3 | 0.2 |
| Kanpur | 0.36 | 0.2 | 11.6 | 1.1 | 0.08 |
| Allahabad | 3.5 | 2.21 | 0.15 | 0.4 | ND |
| Varanasi | 0.7 | 1.9 | 85.4 | 2.2 | 0.1 |
| Patna | 5.0 | ND | 5.03 | 1.17 | ND |
| Bhagalpur | 17.6 | 12.3 | 17.9 | 16.4 | 11.8 |

Table S2. Pesticides reported from different sites along the length of river Ganga.

| Compound  Season | Ʃ endosulfan | Ʃ HCH | Ʃ DDT | Ʃ chlordane | Methoxychlor | Endosulfan sulfate |
| --- | --- | --- | --- | --- | --- | --- |
| Post monsoon | 13.0 | 2266 | 93.0 | 104.0 | 7.0 | 7.0 |
| Post winter | 29.0 | 497.0 | 138.0 | 72.0 | 15.0 | 13.0 |

Table S3. Post monsoon and post winter change in pesticide concentration of river Ganga.

| Compound  Site | Ʃ HCH | Ʃ DDT | Ʃ endosulfan |
| --- | --- | --- | --- |
| Kedar ghat | 15806 | 143226 | 66516 |
| Chousathi ghat | 441 | 49413 | 54628 |
| Mir ghat | ND | 80255 | 28978 |
| Panch ghat | 726 | 321 | 761 |
| Rajendra Prasad ghat | 401 | 21628 | 6284 |
| Ram nagar | 916 | 37544 | 37171 |

Table S4. pesticides reported along different sites of river Ganga.

| Compound  Site | Ʃ HCH | Ʃ DDT | Ʃ endo | Endosulfan sulfate | Aldrin | Heptachlor | Heptachlor epoxide |
| --- | --- | --- | --- | --- | --- | --- | --- |
| Discharge point | 269 | 54 | 54 | 13 | 90 | 519 | 398 |
| Urban | 6 | 3 | 34 | 2 | 11 | 7 | 12 |
| Sub-urban | 6 | 4 | 37 | 2 | 7 | 8 | 14 |

Table S5. Pesticides reported from different sites of river Ganga.

| Compound  Site | Season | Ʃ HCH | Ʃ DDT | Aldrin | Endosulfan |
| --- | --- | --- | --- | --- | --- |
| Buxar | Monsoon | 663 | 291 | 167 | 430 |
| Patna |  | 1160 | 773 | 286 | 14 |
| Mokama |  | 916 | 376 | 1089 | 30 |
| Rajmahal |  | 661 | 337 | 128 | 66 |
| Buxar | Summer | 1240 | 285 | 117 | 29.8 |
| Patna |  | 2200 | 1080 | 79 | 69 |
| Mokama |  | 1200 | 799 | 350 | 80 |
| Rajmahal |  | 912 | 563 | 169 | 134 |
| Buxar | Winter | 1240 | 240 | 119 | 32 |
| Patna |  | 2590 | 1336 | 89 | 68 |
| Mokama |  | 1270 | 837 | 369 | 92 |
| Rajmahal |  | 977 | 374 | 182 | 34 |

Table S6. pesticides reported in three different seasons along different sites of river ganga.
